# A nonsense mutation in *TLR5* is associated with survival and reduced IL-10 and TNF-α levels in human melioidosis

**DOI:** 10.1371/journal.pntd.0005587

**Published:** 2017-05-05

**Authors:** Panjaporn Chaichana, Narisara Chantratita, Florian Brod, Sirikamon Koosakulnirand, Kemajittra Jenjaroen, Suchintana Chumseng, Manutsanun Sumonwiriya, Mary N. Burtnick, Paul J. Brett, Prapit Teparrukkul, Direk Limmathurotsakul, Nicholas P. J. Day, Susanna J. Dunachie, T. Eoin West

**Affiliations:** 1 Mahidol-Oxford Tropical Medicine Research Unit, Mahidol University, Bangkok, Thailand; 2 Department of Microbiology and Immunology, Faculty of Tropical Medicine, Mahidol University, Bangkok, Thailand; 3 Jenner Institute, University of Oxford, Oxford, United Kingdom; 4 Department of Microbiology, Faculty of Science, Mahidol University, Bangkok, Thailand; 5 Department of Microbiology and Immunology, University of South Alabama, Mobile, AL, United States of America; 6 Sappasithiprasong Hospital, Ubon Ratchathani, Thailand; 7 Department of Tropical Hygiene, Faculty of Tropical Medicine, Mahidol University, Bangkok, Thailand; 8 Center for Tropical Medicine and Global Health, University of Oxford, Oxford, United Kingdom; 9 International Respiratory and Severe Illness Center, University of Washington, Seattle, Washington, United States of America; 10 Division of Pulmonary and Critical Care Medicine, Department of Medicine, University of Washington, Seattle, Washington, United States of America; Institut Pasteur, FRANCE

## Abstract

**Background:**

Melioidosis, caused by the flagellated bacterium *Burkholderia pseudomallei*, is a life-threatening and increasingly recognized emerging disease. Toll-like receptor (TLR) 5 is a germline-encoded pattern recognition receptor to bacterial flagellin. We evaluated the association of a nonsense *TLR5* genetic variant that truncates the receptor with clinical outcomes and with immune responses in melioidosis.

**Methodology/Principal findings:**

We genotyped *TLR5* c.1174C>T in 194 acute melioidosis patients in Thailand. Twenty-six (13%) were genotype CT or TT. In univariable analysis, carriage of the c.1174C>T variant was associated with lower 28-day mortality (odds ratio (OR) 0.21, 95% confidence interval (CI) 0.05–0.94, *P* = 0.04) and with lower 90-day mortality (OR 0.25, 95% CI 0.07–086, *P* = 0.03). In multivariable analysis adjusting for age, sex, diabetes and renal disease, the adjusted OR for 28-day mortality in carriers of the variant was 0.24 (95% CI 0.05–1.08, *P* = 0.06); and the adjusted OR for 90-day mortality was 0.27 (95% CI 0.08–0.97, *P* = 0.04). c.1174C>T was associated with a lower rate of bacteremia (*P* = 0.04) and reduced plasma levels of IL-10 (*P* = 0.049) and TNF-α (*P* < 0.0001). We did not find an association between c.1174C>T and IFN-γ ELISPOT (T-cell) responses (*P* = 0.49), indirect haemagglutination titers or IgG antibodies to bacterial flagellin during acute melioidosis (*P* = 0.30 and 0.1, respectively).

**Conclusions/Significance:**

This study independently confirms the association of *TLR5* c.1174C>T with protection against death in melioidosis, identifies lower bacteremia, IL-10 and TNF-α production in carriers of the variant with melioidosis, but does not demonstrate an association of the variant with acute T-cell IFN-γ response, indirect haemagglutination antibody titer, or anti-flagellin IgG antibodies.

## Author summary

Melioidosis is a high-mortality infectious disease in Southeast Asia and northern Australia caused by *Burkholderia pseudomallei*, which is a flagellated, rod-shaped Gram-negative bacterium. Understanding protective host immune responses to melioidosis is fundamental for effective vaccine development. A previous study demonstrated a strong relationship between a *TLR5* stop codon polymorphism that encodes a truncated receptor for bacterial flagellin and protection against death from melioidosis. In this study, we confirmed the relationship of this genetic variant with survival from acute melioidosis in adult patients in northeast Thailand, and identified an association with a lower rate of bacteremia. We also demonstrated that this variant was associated with an increase in peripheral lymphocyte count, but we did not find an association with *B*. *pseudomallei*-specific lymphocyte responses; i.e., IFN-γ secreted T cell response, indirect haemagglutination titers or anti-flagellin IgG antibodies. In addition, patients with the *TLR5* variant have significantly lower levels of IL-10 and TNF-α cytokines in plasma. Our findings further the understanding of the role of TLR5 in protective host immune responses against fatal melioidosis, and inform efforts to develop novel vaccines and therapeutics for melioidosis.

## Introduction

Melioidosis is caused by the Gram-negative, flagellated bacillus and environmental saprophyte, *Burkholderia pseudomallei*, which the US Centers for Disease Control and Prevention (CDC) have identified as a Tier 1 bioterrorism agent. Clinical presentations of melioidosis range from acute sepsis to chronic and persistent infections, and the overall mortality rate can exceed 40% in endemic regions including northeast Thailand [1–3]. Pre-existing conditions such as diabetes, renal disease, excessive alcohol use and increasing age are known risk factors [1,2]. Further expansion of endemic boundaries of melioidosis [4–7], increasing prevalence of diabetes [8], and population ageing [9] lead to an urgent demand for a vaccine against melioidosis, especially in at-risk populations.

Understanding host defense mechanisms against *B*. *pseudomallei* infection is crucial for vaccine design and development, to allow selection of the best vaccine platform including adjuvant, and may drive development of novel therapeutics. Emerging evidence suggests the importance of membrane-bound Toll-like receptors (TLRs) in defense against *B*. *pseudomallei* infection *in vitro* and *in vivo* [10–12], and the TLR5 ligand flagellin has potential as a vaccine adjuvant [13]. Single nucleotide variants (SNV) in TLR genes may influence the innate immune response by altering the magnitude and quality of intracellular signaling cascades with implications for susceptibility to infection and disease outcomes [14]. A recent analysis demonstrated a significant association of the *TLR5* SNV c.1174C>T with protection against organ failure and death in melioidosis [15]. This variant encodes a stop codon at position 392, truncating the receptor in the extracellular domain [16]. c.1174C>T is associated with lower TLR5-mediated innate immune responses *in vitro* and in healthy subjects whose blood was stimulated *ex vivo* [15]. This hypofunction in TLR5 signaling may result in lower immunopathology and in turn a reduction in sepsis-induced organ failure and death. Furthermore, reduced TLR5 signaling could result in lower levels of the regulatory cytokine interleukin-10 (IL-10), leading to less suppression of the host immune defense against the bacteria [15]. However, the relationship between c.1174C>T and innate immune responses has not been studied in patients with melioidosis.

TLRs activate signals crucial for the initiation and modulation of adaptive immune responses such as TLR-dependent dendritic cell control of T-cell activation [17]. Many individuals living in northeast Thailand become seropositive to *B*. *pseudomallei* at a young age, indicating that environmental exposure to the bacterium and the development of adaptive immune responses in the absence of clinical infection is common [18]. A previous study in this cohort reported reduced T-cell responses in patients with acute melioidosis that did not survive [19], raising the possibility that c.1174C>T may protect against death by enhancing T-cell mediated immunity against *B*. *pseudomallei*. Therefore it was important to characterize the association between c.1174C>T and adaptive immune responses in melioidosis.

The objective of this study was to confirm in an independent, prospectively designed cohort the previously reported association of c.1174C>T with survival in acute melioidosis, and to determine whether c.1174C>T is associated with innate and adaptive immune responses in patients with melioidosis.

## Materials and methods

### Ethics statement

The study was approved by the ethics committees of Faculty of Tropical Medicine, Mahidol University (Submission number TMEC 12–014); of Sappasithiprasong Hospital, Ubon Ratchathani (reference 018/2555); and the Oxford Tropical Research Ethics Committee (reference 64–11). The study was conducted according to the principles of the Declaration of Helsinki (2008), and the International Conference on Harmonization (ICH) Good Clinical Practice (GCP) guidelines. Written informed consent was obtained for all patients enrolled in the study.

### Patient cohort

The prospective recruitment of patients with melioidosis for immunological studies at Sappasithiprasong Hospital, Ubon Ratchathani, Thailand has been described previously [19]. Two hundred in-patients aged 18 years or older with melioidosis were enrolled, at a median of 5 days (IQR 3–6, range 2–13) after admission. Melioidosis was defined as isolation of *B*. *pseudomallei* from any clinical sample (blood, sputum, throat, endotracheal, bronchoalveolar lavage, pus, or urine), submitted to the laboratory. HIV status was not tested but previous work in the hospital has shown HIV rates are low and HIV is not a major risk factor for melioidosis [20]. Whole blood samples were collected at the time of enrollment (week 0), as well as again at weeks 12 and 52 after admission to hospital in surviving patients. 194 patients were successfully genotyped and analyzed in this study.

### Genetics methods

Genomic DNA was extracted from blood samples using QIAamp DNA Blood Midi kit (QIAgen, Hilden, Germany) according to the company’s instruction and stored at -20°C. The *TLR5* c.1174C>T (rs5744168) SNV was genotyped using TaqMan® SNP genotyping assay (Applied Biosystems, CA, USA) on a CFX96 Touch Real-Time PCR Detection System (BioRad, Hercules, USA). The SNV context sequence was TGAATGGTTGTAAGAGCATTGTCTC**[A/G]**GAGATCCAAGGTCTGTAATTTTTCC.

### *Ex vivo* interferon-γ (IFN-γ) Enzyme-linked immunosorbent spot-forming cell assay (ELISPOT)

The magnitude of cellular responses to *B*. *pseudomallei* was determined by *ex vivo* IFN-γ ELISPOT assay, as previously described [19]. Briefly, 96-well Multiscreen-I plates (Millipore, UK) were coated with 1D1K anti-human IFN-γ (Mabtech, AB, Sweden) and stored at 4°C overnight. Fresh peripheral blood mononuclear cells (PBMC) at 2 x 10^5^ cells per well were added in duplicate and whole heat-inactivated *B*. *pseudomallei* (HIA-Bp) clinical isolates 199a and 207a [21] at concentration of 20 μg/ml were then added. Phytohemagglutinin (PHA) at final concentration of 5 μg/ml and RPMI-1640 were used as positive and negative controls, respectively. A T cell peptide pool (CEF, (Mabtech) at concentration of 1 μg/ml was used as control antigens. After 18 hours, secreted IFN-γ was detected following the manufacturer’s protocol (Mabtech) and read under CTL ELISPOT reader. Results are expressed as IFN-γ spot-forming cells (SFC) per million PBMC.

### Indirect haemagglutination assay (IHA)

Titers of antibodies against *B*. *pseudomallei* were assessed by IHA following a standard protocol at the Mahidol-Oxford Tropical Medicine Research Unit, as modified from a protocol previously described [18,22]. Briefly, two-fold dilutions of patient serum were added to 96-well U bottom microplate containing 25 μl of HIA-Bp-sensitized sheep red blood cells. Plates were left at room temperature for 2 hours before incubation at 4°C overnight. The results were recorded as the highest dilution when a positive reaction was observed. The cut off was set at a dilution of 1:40.

### *B*. *pseudomallei* flagellin antibody assay

Plasma levels of IgG antibodies specific to flagella of *B*. *pseudomallei* were determined by rapid Enzyme-Linked Immunosorbant Assay (ELISA), as described in a previous study [23] using recombinant flagellin (rFliC) as the coating antigen. The *fliC* gene (BPSL3319) was PCR amplified from *B*. *pseudomallei* K96243 genomic DNA, cloned into pBAD/HisA (Invitrogen, USA) and expressed in *E*. *coli* as previously described [24].

To perform ELISA, the purified rFliC antigen was added to wells of a 96-well U-bottom immunoplate (Nunc MaxiSorp U-bottom 96-Well plates; Thermo Scientific, Denmark) at a concentration of 15 μg/ml and incubated overnight at 4°C. Between each step, the ELISA plate was washed with 0.05% Tween-20 in PBS 4 times. After blocking at 37°C for 2 hours with 5% skim milk in PBS, patients’ plasma was diluted 1:300 and added to the pre-coated ELISA plate in duplicate then incubated at room temperature for 2 hours. The secondary antibody, HRP-conjugated rabbit antihuman IgG (DAKO, Copenhagen, Denmark), was diluted 1:2000 then added to the plate and incubated for 30 minutes. ELISAs were developed using TMB substrate. Results were determined as absorbance value (OD_450_). Pooled plasma from five melioidosis patients and five healthy controls were used as positive and negative controls, respectively.

### Cytokine assays

Heparinized plasma for immunoassays was separated from blood by density centrifugation within three hours of blood draw. Cytokine levels in the plasma were quantified by using ELISA kits according to manufacturers’ instructions; The Human IL-10 and TNF-α Instant ELISA kits (eBioscience, San Diego, CA, USA), human granulocyte colony-stimulating factor (G-CSF) ELISA kit (Abcam, Cambridge, MA, USA), and human transforming growth factor beta 1 (TGF-β1) DuoSet ELISA kit (R&D systems, Minneapolis, MN, USA). Concentrations of cytokines were calculated from standard curves.

### Statistics

Categorical variables were displayed as counts and proportions, and were compared using Pearson’s chi squared test or Fisher’s exact test. Non-normally distributed continuous data were reported as median and interquartile range (IQR). The significance of differences between two groups was analyzed by Mann-Whitney U-test in Graphpad Prism Version 6 (San Diego, USA). In addition, immunological data was divided into tertiles and the distribution of the *TLR5* genotype was compared between the highest third and lowest third of responses by Mann-Whitney U-test. To test the association of genotype with outcome, we performed univariable logistic regression and multivariable logistic regression adjusting for age, sex, diabetes and pre-existing renal disease using Stata version 14.0 for Window (StataCorp LP, TX, USA). Survival analysis was assessed with log-rank test of Kaplan-Meier curve by Stata version 11.1. A *P* value <0.05 was considered significant.

## Results

### *TLR5* c.1174C>T is associated with survival and reduced bacteremia in acute melioidosis patients

To confirm the previously reported association of the *TLR5* variant c.1174C>T (rs5744168) with protection against death in acute melioidosis patients, we genotyped the variant in 194 Thai patients with culture-proven melioidosis admitted at Sappasithiprasong Hospital. Of these, 168 (86.6%) were genotype CC, 25 (12.8%) were CT and one (0.5%) was TT. The characteristics of the melioidosis patient cohort have been described in detail elsewhere [19], with key clinical information including demographics and risk factors shown in Table 1. Despite receiving appropriate antibiotic treatment, 25.3% (49/194) of patients died within 28 days of admission to hospital (28-day mortality). A further 12 patients died between days 29 and 90 after admission resulting in a 90-day mortality rate of 31.4% (61/194).

**Table 1:** Patients’ characteristics and mortality.

	**All (n = 194)**	**28-day mortality**			**90-day mortality**		
		Non-survivors (n = 49)	Survivors (n = 145)	*P-value*^a^	Non-survivors (n = 61)	Survivors (n = 133)	*P-value*^a^
Baseline characteristics							
Age (median with IQR)	56 (46–63)	58 (49–69.5)	54 (46–61)	0.01	59 (49–68.5)	54 (45.5–59.5)	0.002
Sex (male)	129 (66%)	30 (61%)	99 (68%)	0.37	38 (62%)	91 (68%)	0.40
Sex (female)	67 (34%)	19 (39%)	46 (32%)	0.37	23 (38%)	42 (32%)	0.40
Pre-existing conditions							
Diabetes	112 (58%)	24 (49%)	88 (61%)	0.37	38 (62%)	91 (68%)	0.40
Chronic liver disease	7 (4%)	3 (6%)	4 (3%)	0.37	3 (5%)	4 (3%)	0.68
Renal disease	34 (17.5%)	14 (29%)	20 (14%)	0.02	17 (28%)	17 (13%)	0.01
Heart disease	23 (12%)	7 (14%)	16 (11%)	0.61	11 (18%)	12 (9%)	0.07
Previous melioidosis	5 (2.5%)	1 (2%)	4 (3%)	1.00	2 (3%)	3 (2%)	0.65
Clinical presentations							
Bacteremia	99 (51%)	37 (75.5%)	62 (43%)	< 0.001	45 (74%)	54 (41%)	< 0.001
Pneumonia	48 (25%)	15 (31%)	33 (23%)	0.27	18 (29.5%)	30 (22.5%)	0.30

^a^ For categorical variables, *P values* were determined with Pearson’s chi squared test or Fisher’s exact test for cells with value <10. For continuous variables, *P values* were determined with the Mann-Whitney U-test.

We confirmed Hardy–Weinberg equilibrium in survivors (*P* = 1) before testing the association of c.1174C>T with mortality. When 28-day mortality was selected as outcome, 16.6% of survivors were CT or TT genotypes, whereas 4.1% of non-survivors were these genotypes (*P* = 0.055, Table 2). We also observed the same pattern in analysis of 90-day mortality: 17.3% of survivors were heterozygotes or minor homozygotes, compared with 4.9% of non-survivors (*P* = 0.03). In a dominant genetic model (combining CT and TT subjects into the same group), the c.1174C>T variant was significantly associated with survival at both 28 days and 90 days [odds ratio (OR) for death 0.21, 95% Confidence Interval (CI) 0.05–0.94, *P* = 0.04 for 28-day mortality, and OR 0.25, 95% CI 0.07–086, *P* = 0.03 for 90-day mortality].

**Table 2:** Crude association of *TLR5* c.1174C>T with mortality and bacteremia.

	**Genotype**	**General Genetic Model**			**Dominant Model**		
		Yes	No	*P-value*	OR	95%CI	*P-value*
28-day mortality							
	**CC**	47 (95.9%)	121 (83.5%)				
	**CT**	2 (4.1%)	23 (15.9%)	0.055	0.21	0.05–0.94	0.04
	**TT**	0 (0%)	1 (0.7%)				
90-day mortality							
	**CC**	58 (95.1%)	110 (82.7%)				
	**CT**	3 (4.9%)	22 (16.5%)	0.03	0.25	0.07–0.86	0.03
	**TT**	0 (0%)	1 (0.8%)				
Bacteremia							
	**CC**	91 (91.9%)	77 (81%)				
	**CT**	8 (8.1%)	17 (17.9%)	0.04	0.38	0.15–0.91	0.03
	**TT**	0 (0%)	1 (1.1%)				

**Fig 1:**
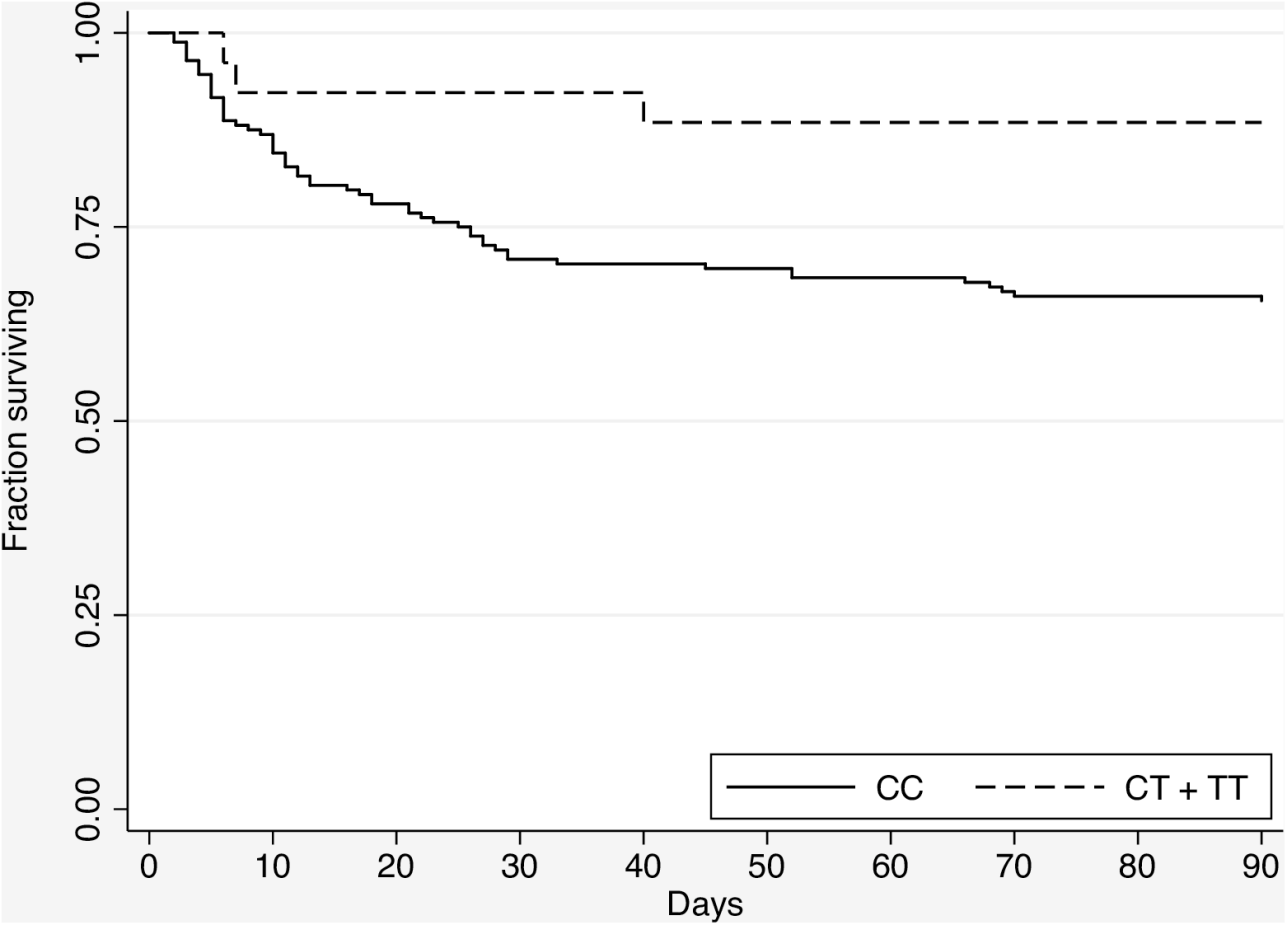
Kaplan-Meier survival curve of melioidosis subjects demonstrated enhanced survival in carriers of *TLR5* c.1174C>T. Curves are significantly different by the log rank test (*P = 0*.*03*).

We also plotted the Kaplan Meier survival curve by c.1174C>T genotype for melioidosis subjects (Fig 1). The risk of death for subjects carrying the CC genotype was significantly higher than those carrying CT or TT genotypes by the log-rank test (*P* = 0.03).

We next evaluated the association between the c.1174C>T variant and bacteremia. In this cohort of melioidosis patients, 99 patients (51%) had bacteremia and bacteremia was tightly associated with mortality (*P* < 0.0001, Table 1) [19]. Thus, bacteremia can be considered an intermediate outcome measure for control of *B*. *pseudomallei*. Our results in the present study show that 8.1% (8/99) of patients with bacteremia were CT or TT genotypes, compared with 19.0% (18/95) of patients who had no bacteremia (*P* = 0.04, Table 2). We found that the c.1174C>T variant was associated with a lower rate of bacteremia in an unadjusted dominant model (OR for bacteremia 0.38, 95% CI 0.15–0.91, *P* = 0.03). Taken together, these results demonstrate a decrease in fatality and bacteremia in patients carrying the *TLR5* c.1174C>T variant compared to those without the variant.

### Association between *TLR5* c.1174C>T and survival in multivariable adjusted model

To take into account other drivers of immune responses to bacterial infection, we next tested the association of the c.1174C>T variant with death using an adjusted multivariable model including potential confounding variables. The odds ratio point estimates of the effect of c.1174C>T on mortality did not change appreciably from the univariable model. The c.1174C>T variant showed a borderline evidence of an association with 28-day mortality (*P* = 0.06) but remained significantly associated with 90-day mortality (*P* = 0.04) and bacteremia (*P* = 0.04) when sex, age and the major pre-existing conditions of diabetes and renal disease were incorporated into the model (S1 Table).

We also found a significant association between increasing age and 28-day mortality (OR 1.037 95% CI 1.009–1.066 for each year of age) and 90-day mortality (OR 1.030 95% CI = 1.012–1.067 for each year of age) in the multivariable-adjusted logistic regression model. In our cohort, the odds of bacteremia for melioidosis patients with pre-existing renal disease were 3.7 times higher than for those without renal disease (*P* = 0.003, S1 Table). We did not observe any significant association gender or diabetes and 28-day mortality, 90-day mortality or bacteremia.

### *TLR5* c.1174C>T is associated with lymphocyte count but not with T-cell IFN-γ response and IHA titer to *B*. *pseudomallei*

We then examined the relationship between c.1174C>T and absolute neutrophil and lymphocyte counts during acute melioidosis. As shown in Table 3, the median peripheral blood neutrophil count of patients with the CC genotype was comparable to that of patients with CT or TT genotypes (*P* = 0.87). In contrast, absolute lymphocyte counts in patients with the CC genotype were significantly lower than those of patients with CT or TT genotypes (Table 3; *P* = 0.02).

**Table 3:** Immune response measurements by *TLR5* c.1174C>T genotype.

**Measurement**	**CC** **Median (IQR)**	**CT + TT** **Median (IQR)**	***P-value***^a^
Neutrophil count	9513 (6486–12 575)	8588 (5608–14 713)	0.87
Lymphocyte count / μl	1282 (795–1902)	1785 (1295–2408)	0.02
T-cell response to T cell peptide pool (CEF), by IFN-γ ELISPOT (SFC / 10^6^ PBMC)	18.8 (1–327.5)	160 (1–1157.5)	0.32
T-cell response to *B*. *pseudomallei*, by IFN-γ ELISPOT (SFC / 10^6^ PBMC)	25 (3–90)	24 (6–113)	0.49
Antibody response to *B*. *pseudomallei*, titer by IHA	160 (40–1280)	80 (18–640)	0.30
Anti-FliC IgG antibody response, OD_450_ by ELISA	0.53 (0.23–1.45)	0.92 (0.42–1.45)	0.10
Plasma IL-10 By ELISA (pg / ml)	17.0 (6.0–35.2)	8.6 (0.34–21.74)	0.049
Plasma G-CSF By ELISA (pg / ml)	3.0 (0.0–56.2)	20.1 (0.0–38.9)	0.83
Plasma TNF-α By ELISA (pg/ml)	4.7 (0.6–7.7)	0.0 (0.0–2.7)	<0.0001
Plasma TGF-β1 by ELISA (ng/ml)	7.7 (4.9–10.35)	6.9 (3.9–9.5)	0.21

SFC / 10^6^ PBMC = spot forming cells per million peripheral blood mononuclear cells. IHA = indirect haemagglutination assay

^a^Mann-Whitney U-test

T cell responses as quantified by IFN-γ ELISPOT and IHA titers to *B*. *pseudomallei* are lower in patients with melioidosis who do not survive [19]. In the previous study, we found undetectable or very low mean IFN-γ ELISPOT response in healthy controls (15 SFC per million PBMC) when compared with melioidosis patients (133 SFC per million PBMC) [19]. We therefore evaluated the association of c.1174C>T with these adaptive immune responses to *B*. *pseudomallei* in melioidosis patients. At the time of enrollment with acute melioidosis (median day 5 of hospitalization), the median IFN-γ ELISPOT response of subjects with the CC genotype was not different from those having CT or TT genotypes after stimulation with either the T-cell control peptide pool CEF (*P* = 0.32) or heat-killed *B*. *pseudomallei* (*P* = 0.49; Table 3, S1A and S1B Fig).

When subjects’ responses were grouped in tertiles according to IFN-γ ELISPOT results, we did not observe any difference in low (≤ 7.5 SFC/10^6^ PBMC) and high responders (≥65 SFC/10^6^ PBMC) by genotype. The median IFN-γ ELISPOT result of low responder with CC and CT or TT genotypes was 1 (IQR 1–2.5) and 3 (IQR 1–5), respectively (*P = 0*.*47*); and those result of high responder with CC and CT or TT genotypes was 201.25 (IQR 92.5–380) and 262.5 (IQR 105–625), respectively (*P = 0*.*43*).

We assessed the relationship of c.1174C>T with IHA titer. There was no difference based on genotype as the median IHA titer at the time of enrollment of patients carrying CC was 160 (IQR 40 to 1280) and those of individuals carrying CT or TT was 80 (IQR 18 to 640, *P* = 0.30; Table 3 and S1C Fig). Grouping the IHA titer by low (titer ≤ 1:160) and high responder (titer > 1: 160) status also did not demonstrate any relationship with genotype. Likewise, plasma levels of IgG antibodies to *B*. *pseudomallei* flagellin, a known ligand of TLR5, (anti-FliC), obtained at enrollment, were not statistically significantly different between melioidosis patients with CC (median 0.53, IQR 0.23–1.45) and CT or TT genotypes (median 0.92, IQR 0.42–1.45, *P* = 0.10, Table 3 and S1D Fig). We also found that plasma anti-FliC antibody levels were not significantly different between survivors (median 0.57, IQR 0.25–1.37) and fatal cases (median 0.48, IQR 0.26–1.59, *P = 0*.*64*).

We also evaluated the relationship between c.1174C>T and the kinetics of the T-cell IFN-γ response and IHA titer over one year (sample were collected at week 0, 12 and 52). We did not see a significant difference of kinetics in IFN-γ ELISPOT response between these two patients groups (S2A Fig). However, we found that the median IHA titer of patients with CT or TT genotypes (median 10.5, IQR 1–160) was significantly lower than those having CC genotype (median 320, IQR 80–640) at week 12 after admission (*P* < 0.001) but not at the other time points (S2B Fig).

Although we found a reduced IHA titer in survivors of melioidosis with CT or TT genotypes at week 12 after admission, the results in the present study do not demonstrate an effect of c.1174C>T on the measured T-cell IFN-γ response, IHA titer, or plasma levels of anti-FliC IgG antibodies during the acute phase of *B*. *pseudomallei* infection.

### *TLR5* c.1174C>T is associated with reduced plasma IL-10 and TNF-α levels in melioidosis patients

Stimulation of whole blood with *B*. *pseudomallei* has been previously shown to induce lower levels of monocyte-normalized IL-10 and granulocyte colony-stimulating factor (G-CSF) in healthy individuals with CT or TT genotypes [15]. To determine whether this association of c.1174C>T with experimentally induced inflammatory responses holds during acute melioidosis, we measured the levels of these two cytokines in the plasma of melioidosis patients in our cohort. IL-10 levels were significantly lower in carriers of CT or TT (Table 3 and S3A Fig; median 8.6, IQR 0.34 to 21.74) than in those with CC (median 17.0, IQR 6.0 to 35.2, *P* = 0.049). However, we did not observe a difference of plasma G-CSF levels by genotype (Table 3 and S3B Fig; *P* = 0.83).

We next assayed plasma levels of the pro-inflammatory cytokine TNF-α that has been previously associated with death in melioidosis [25]. We found that plasma TNF-α levels in patients with the CC genotype (Table 3 and S3C Fig; median 4.73, IQR 0.605 to 7.66) were significantly higher than those having CT or TT genotypes (mostly undetectable). In addition, the patients with bacteremia also showed a trend toward higher levels of TNF-α in plasma (median 3.65, IQR 0–7.28) compared with those with no bacteremia (median 0.53, IQR 0–4.51, *P* = 0.06). We also measured plasma levels of transforming growth factor beta 1 (TGF-β1), another immunoregulatory cytokine, but we did not observe a significant difference between patients with CC compared to CT or TT genotypes (Table 3 and S3D Fig; *P* = 0.21).

## Discussion

Data in this study confirm a previous report [15] demonstrating a significant association between the nonsense *TLR5* c.1174C>T variant and survival in melioidosis patients. We also identified a relationship between c.1174C>T and lower rates of bacteremia, which represents improved control of the infection. These data underscore the importance of TLR5-dependent signaling in driving clinical outcomes in human melioidosis. In melioidosis patients, c.1174C>T was also associated with lower plasma levels of both pro-inflammatory cytokine TNF-α and anti-inflammatory cytokine IL-10, implicating differential activation of innate immunity in the mechanism of increased survival attributable to c.1174C>T. In this population with likely broad subclinical exposure to *B*. *pseudomallei* and the development of adaptive immunity, suppressed T-cell responses to *B*. *pseudomallei* are associated with death from acute melioidosis [19]. However, we did not find evidence that the *TLR5* c.1174C>T variant drives the T-cell IFN-γ response, IHA titer, or anti-FliC antibody response during acute melioidosis, suggesting that the mechanism of enhanced survival in carriers of the TLR5 variant may be independent of these adaptive immunological responses.

Our finding of an inhibitory effect of c.1174C>T on IL-10 production in acute melioidosis extends findings from a previous study [15] in which blood from healthy individuals carrying the c.1174C>T variant released less IL-10 upon stimulation with *B*. *pseudomallei*. Together these data suggest a possible role for TLR5-driven IL-10 release in modulating risk of death in melioidosis [25,26]. Low concentrations of IL-10 in plasma may diminish suppressive activity of immune responses, resulting in augmentation of pro-inflammatory activity, and control of bacterial infection. However, our study does not establish causation, and more in-depth investigation is required to clarify the mechanism of TLR5-dependent IL-10 function in melioidosis.

In contrast to whole blood stimulation studies in healthy subjects [15], we found significantly lower levels of plasma TNF-α in melioidosis patients carrying c.1174C>T. This result agrees with studies in rheumatoid arthritis and Salmonella infections, in which flagellin-induced TLR5 ligation leads to upregulation of TNF-α in monocytes or macrophages [27–29]. TNF-α plays a key role in neutrophil recruitment in the inflammatory response to infections; nevertheless, it can also enhance bacterial growth [30,31]. Interestingly, we also found a strong trend towards higher plasma TNF-α levels and the presence of bacteremia. As bacteremia is tightly linked with death, this is consistent with a previous study reporting increased TNF-α levels in non-survivors of melioidosis [25]. In our study, carriers of c.1174C>T had no effect on G-CSF or TGF-β1 production. It is postulated that the release of these cytokines might pass through or compensate by other pathways.

TLR5 plays a critical role in connecting innate and adaptive immunity in other bacterial infections. Accumulating evidence demonstrates that flagellin ligation of TLR5 can simultaneously initiate MyD88-dependent and Spleen tyrosine kinase (Syk)-dependent pathways leading to pro-inflammatory cytokine secretion and antigen presentation to flagellin-specific CD4 T cells, respectively [32–34]. TLR5 activation can also lead to suppression of adaptive immune responses by pathways involving IL-10 as discussed, myeloid-derived suppressor cells (MDSC) [35] and regulatory T-cells (Treg) [36]. However, we did not observe an association between the *TLR5* c.1174C>T genotype and IFN-γ secreting *B*. *pseudomallei*-specific T-cell responses nor serum anti-*B*. *pseudomallei* antibody titers during the acute stage (week 0) of bacterial infection in this study.

The stimuli used in the assays of adaptive immunity in this study may have induced too broad a response to identify the distinct downstream responses of c.1174C>T variant. The T-cell IFN-γ response and IHA titer in our study were assessed using heat-killed whole-cell *B*. *pseudomallei*, which contains a large number of immunogenic antigens. Many bacterial antigens including lipopolysaccharide (LPS) and acyl hydroperoxide reductase (AhpC) can elicit both B- and T-cell responses via other immunogenic pathways besides TLR5 [37,38].

We observed comparable plasma levels of anti-FliC IgG between patients with CC and CT or TT genotypes during acute melioidosis. *Sanders et al* [39] demonstrated that *Salmonella* flagellin elicits a strong IgG response in TLR5^-/-^ mice, indicating that TLR5 is not required for antibody responses to flagellin. Therefore, it is postulated that *B*. *pseudomallei* flagellin may also promote humoral immunity via a TLR5-independent pathway similar to that reported during *Salmonella* infection.

Although we did not see a relationship between TLR5 genotype and IHA titer during acute melioidosis, we found an association between c.1174C>T variant and reduced IHA titer in survivors during convalescence from disease (12 weeks after admission). This could be due to the impact of TLR5 engagement on antibody production and secretion of terminally differentiated plasma cells compared with B-cells at an earlier maturation stage [40]. However, further study on the detailed mechanism of TLR5 triggering on memory B cells is required.

The *TLR5* c.1174C>T variant was associated with a higher absolute lymphocyte count, but not with T-cell responses. The increased number of lymphocytes in patients carrying the variant may result from the reduced suppressive effect of IL-10 induced during acute melioidosis. Further studies should aim to characterize this increased lymphocyte population with a particular focus on B-cells, NK cells or subsets of T cells that do not produce IFN-γ. In our study, the *TLR5* c.1174C>T variant did not influence the quantity of neutrophils in melioidosis patients.

*TLR5* c.1174C>T might play a critical role only in inflammatory cytokine responses against melioidosis, contributing to control of bacterial infection before adaptive immunity takes place. Otherwise, the relationship between *TLR5* c.1174C>T and adaptive immunity may be present but our study had insufficient power or did not measure the relevant T-cell or antibody response. Additional studies focusing on the relationship between the *TLR5* c.1174C>T and adaptive immune responses against *B*. *pseudomallei* flagellin may uncover an association. Further studies will address the direct and crucial link between innate and adaptive immunity of TLR5 in *B*. *pseudomallei*.

In summary, the results of our study provide critical confirmation of the association of *TLR5* c.1174C>T genotype with protection against death in acute melioidosis patients. Our results also suggest that the genotype c.1174C>T in melioidosis patients is associated with reduced production of both pro-inflammatory cytokine TNF-α and anti-inflammatory cytokine IL-10 at the early stage of infection. Although *TLR5* genotype is associated with protection against melioidosis, other factors underlying host defense mechanisms merit exploration in further studies.

## Supporting information

S1 Fig Comparison of T- and B-cell responses in melioidosis by *TLR5* c.1174C>T genotype. Peripheral blood mononuclear cells (PBMC) collected at week 0 were stimulated with T cell peptide pool, CEF (A) or heat-killed *B*. *pseudomallei* (B) for 18 hours and then IFN-γ secreting cells were counted and expressed as spot forming cells per million PBMC (SFC/106 PBMC). (C) Level of antibodies against *B*. *pseudomallei* in patient sera was measured by IHA and expressed as reciprocal of IHA titer. (D) Plasma level of anti-flagellin (FliC) IgG antibodies were measured by ELISA and expressed by OD_450_. All data are shown as median ± interquartile range. n = 167 (CC), n = 24 (CT and TT). *P*-values were determined by Mann-Whitney U-test analysis.
(TIF)

S2 Fig Comparison of kinetics of T- and B-cell responses at week 0, 12 and 52 of melioidosis by *TLR5* c.1174C>T genotype. Peripheral blood mononuclear cells (PBMC) collected at week 0, 12 and 52 after disease onset were stimulated with heat-killed *B*. *pseudomallei* (A) for 18 hours and then IFN-γ secreting cells were counted and expressed as spot forming cells per million PBMC (SFC/10^6^ PBMC). (B) Level of antibodies against *B*. *pseudomallei* in patient sera collected at the same time points was measured by IHA and expressed as reciprocal of IHA titer. All data are shown as median ± interquartile range. n = 167 (CC), n = 24 (CT and TT). *P*-values were determined by Mann-Whitney U-test analysis.
(TIF)

S3 Fig Comparison of pro-inflammatory cytokine profiles in melioidosis by TLR5 c.1174C>T genotype. Quantitative measurement of cytokines IL-10 (A), G-CSF (B), TNF-α (C) and TGF-β1 (D) in patients’ plasma was performed by ELISA. The concentration of cytokines was calculated from standard curves and expressed as median ± interquartile range (IQR) of picogram per milliliter for all cytokines, except for TGF-β-1 which was expressed as nanogram per milliliter. All tests were performed in duplicate. n = 37 (CC), n = 26 (CT and TT). *P*-values were determined by Mann-Whitney U-test analysis.
(TIF)

S1 Table Multivariable-adjusted logistic regression for mortality and bacteremia. (DOCX)
