# Supplementary figures and images for: A nonsense mutation in TLR5 is associated with survival and reduced IL-10 and TNF-α levels in human melioidosis
Source: PLoS Negl Trop Dis. 2017 May 5;11(5):e0005587. doi: 10.1371/journal.pntd.0005587 (PMC5435357; doi:10.1371/journal.pntd.0005587)

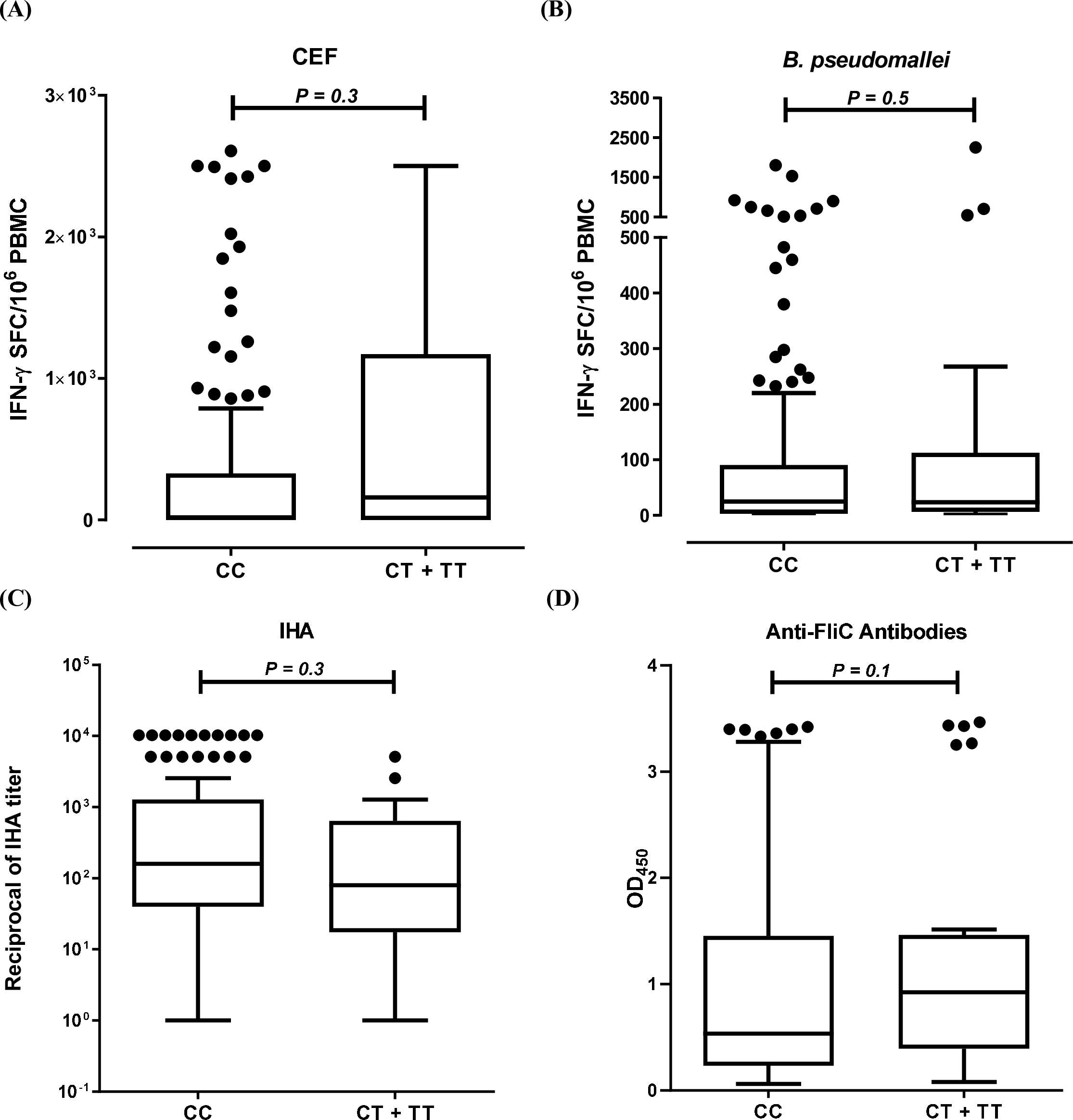

Supplement: S1 Fig — Peripheral blood mononuclear cells (PBMC) collected at week 0 were stimulated with T cell peptide pool, CEF (A) or heat-killed B. pseudomallei (B) for 18 hours and then IFN-γ secreting cells were counted and expressed as spot forming cells per million PBMC (SFC/106 PBMC). (C) Level of antibodies against B. pseudomallei in patient sera was measured by IHA and expressed as reciprocal of IHA titer. (D) Plasma level of anti-flagellin (FliC) IgG antibodies were measured by ELISA and expressed by OD450. All data are shown as median ± interquartile range. n = 167 (CC), n = 24 (CT and TT). P-values were determined by Mann-Whitney U-test analysis. (TIF) [file pntd.0005587.s001.tif]

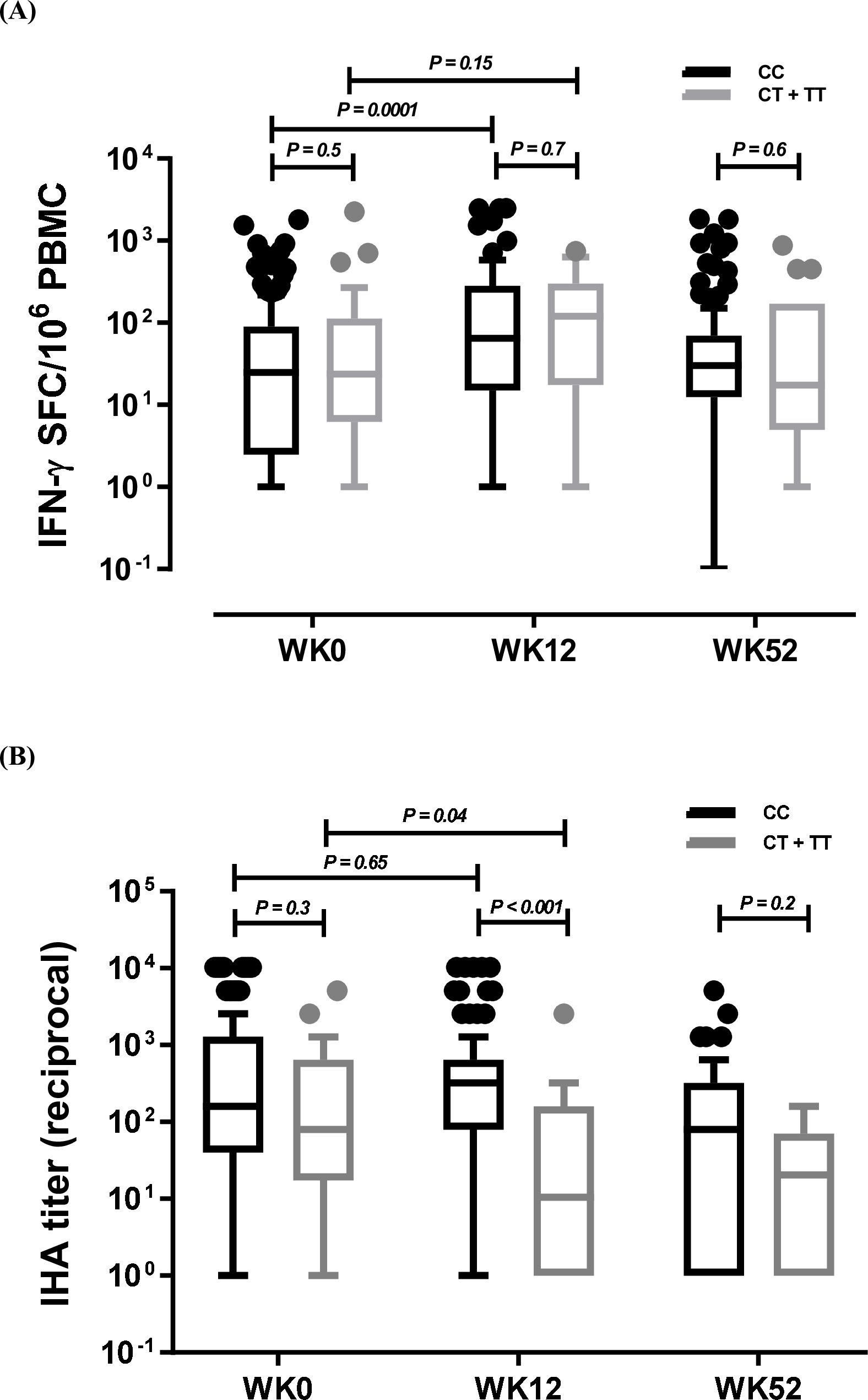

Supplement: S2 Fig — Peripheral blood mononuclear cells (PBMC) collected at week 0, 12 and 52 after disease onset were stimulated with heat-killed B. pseudomallei (A) for 18 hours and then IFN-γ secreting cells were counted and expressed as spot forming cells per million PBMC (SFC/106 PBMC). (B) Level of antibodies against B. pseudomallei in patient sera collected at the same time points was measured by IHA and expressed as reciprocal of IHA titer. All data are shown as median ± interquartile range. n = 167 (CC), n = 24 (CT and TT). P-values were determined by Mann-Whitney U-test analysis. (TIF) [file pntd.0005587.s002.tif]

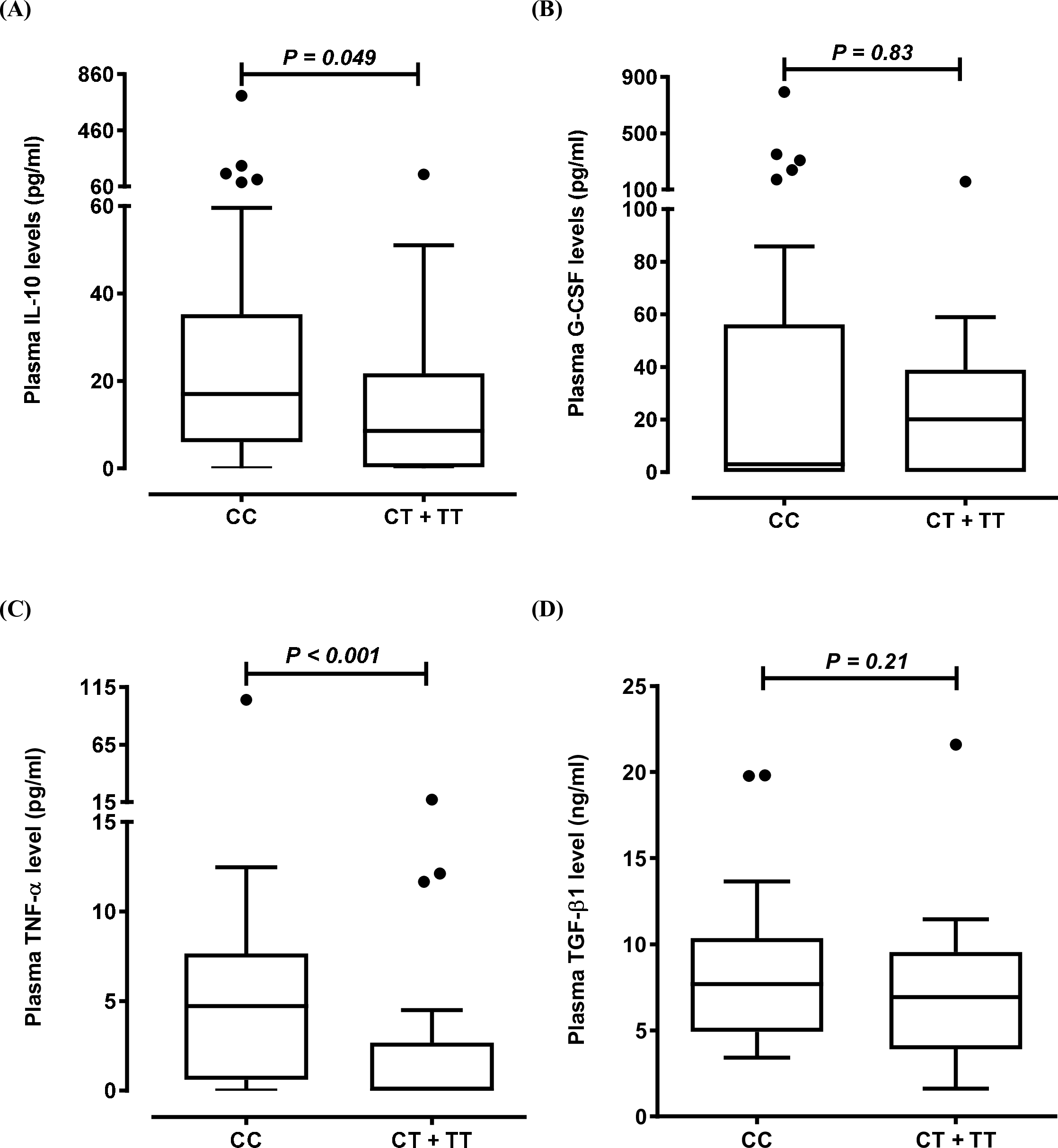

Supplement: S3 Fig — Quantitative measurement of cytokines IL-10 (A), G-CSF (B), TNF-α (C) and TGF-β1 (D) in patients’ plasma was performed by ELISA. The concentration of cytokines was calculated from standard curves and expressed as median ± interquartile range (IQR) of picogram per milliliter for all cytokines, except for TGF-β-1 which was expressed as nanogram per milliliter. All tests were performed in duplicate. n = 37 (CC), n = 26 (CT and TT). P-values were determined by Mann-Whitney U-test analysis. (TIF) [file pntd.0005587.s003.tif]
