# Supplementary material for: A nonsense mutation in TLR5 is associated with survival and reduced IL-10 and TNF-α levels in human melioidosis
Source: PLoS Negl Trop Dis. 2017 May 5;11(5):e0005587. doi: 10.1371/journal.pntd.0005587 (PMC5435357; doi:10.1371/journal.pntd.0005587)
Supplement: S1 Table — (DOCX) [file pntd.0005587.s004.docx]

**S1 Table. Multivariable-adjusted logistic regression for mortality and bacteremia**

| Variables | 28-day mortality | | 90-day mortality | | Bacteremia | |
| --- | --- | --- | --- | --- | --- | --- |
|  | **Adjusted OR (95% CI)** | ***P-value*** | **Adjusted OR (95% CI)** | ***P-value*** | **Adjusted OR (95% CI)** | ***P-value*** |
| c.1174C>T | 0.241 (0.054-1.076) | 0.06 | 0.274 (0.078-0.966) | 0.04 | 0.390 (0.156-0.975) | 0.04 |
| Sex (male = 1) | 0.546 (0.259-1.149) | 0.11 | 0.558 (0.274-1.136) | 0.11 | 1.015 (0.527-1.955) | 0.96 |
| Age | 1.037 (1.009-1.066) | 0.01 | 1.039 (1.012-1.067) | 0.005 | 1.023 (0.999-1.048) | 0.06 |
| Diabetes | 0.0870 (0.421-1.798) | 0.71 | 0.913 (0.459-1.815) | 0.79 | 1.698 (0.887-3.252) | 0.11 |
| Pre-existing renal disease | 2.08 (0.883-4.4564) | 0.10 | 2.146 (0.964-4.778) | 0.06 | 3.728 (1.544-8.997) | 0.003 |
